# Plummer‐Vinson Syndrome With Coexistent Thyro‐Cardiac Disease and Acute Decompensated Heart Failure: A Case Report

**DOI:** 10.1002/ccr3.70447

**Published:** 2025-04-16

**Authors:** Suleiman Ayalew, Michael A. Negussie, Tinsae Anebo, Fitsum A. Gemechu, Yishak Abdulsemed, Segenet B. Mengistu

**Affiliations:** ^1^ School of Medicine, College of Medicine and Health Sciences University of Gondar Gondar Ethiopia; ^2^ School of Medicine, College of Health Sciences Addis Ababa University Addis Ababa Ethiopia; ^3^ Department of Internal Medicine Thomas Jefferson University—Sidney Kimmel Medical College Philadelphia Pennsylvania USA; ^4^ Department of Internal Medicine College of Medicine and Health Sciences, University of Gondar Gondar Ethiopia

**Keywords:** case report, esophageal web, Plummer‐Vinson syndrome, thyro‐cardiac disease

## Abstract

Plummer‐Vinson Syndrome (PVS) is a rare disorder characterized by the triad of dysphagia, iron‐deficiency anemia, and esophageal webs. Limited data on its prevalence exist, with most information derived from case reports. We present the case of a 60‐year‐old woman with a 6‐month history of a progressively enlarging anterior neck mass and worsening symptoms of heart failure, heat intolerance, and dysphagia. Diagnostic evaluation revealed a multinodular goiter, microcytic hypochromic anemia, hyperthyroidism, hypokalemia, and esophageal webs. She was diagnosed with PVS in conjunction with thyro‐cardiac disease and acute decompensated heart failure. Treatment included iron supplementation, pneumatic dilation, and management of her thyroid and cardiac conditions, resulting in significant clinical improvement. This case demonstrates the importance of early recognition and treatment of PVS to prevent complications and reduce morbidity.

**Summary**

- Early recognition of Plummer‐Vinson syndrome in patients presenting with dysphagia and iron‐deficiency anemia is essential to prevent serious complications.
- Timely diagnosis becomes even more important when PVS coexists with complex conditions such as thyro‐cardiac disease and acute heart failure.
- In such cases, a multidisciplinary approach is essential to ensure comprehensive and effective management.

## 1 Introduction

Plummer‐Vinson syndrome (PVS), also known as Paterson‐Brown‐Kelly syndrome, is an extremely rare condition characterized by the triad of dysphagia, iron‐deficiency anemia, and esophageal webs. Data on the incidence and prevalence of PVS is scarce due to its rarity, with the majority of available literature being composed of case reports and small case series [1]. This syndrome predominantly affects middle‐aged women and is associated with an increased risk of esophageal squamous carcinoma [1, 2]. This case report details the presentation, diagnostic workup, and management of a 60‐year‐old female who was diagnosed with PVS alongside thyro‐cardiac disease and acute decompensated heart failure.

## 2 Case History and Physical Examination

A 60‐year‐old female with no significant prior medical history presented with a painless swelling in the anterior neck, first noted 6 months ago. The swelling had been progressively increasing in size since its onset. Five months ago, she started feeling easily fatigued, along with heat intolerance, tremors, and palpitations. These symptoms persisted and worsened, but she did not seek medical attention. Two months later, she developed difficulty swallowing both solid and liquid foods, which steadily became more severe. This was accompanied by dizziness, significant weight loss, and episodes of epigastric pain. Three weeks prior to her current presentation, her condition took a marked turn for the worse. She experienced shortness of breath at rest and orthopnea, which required her to sleep with two pillows. Additionally, she noticed swelling in her legs up to the ankles, a productive cough with whitish frothy sputum, and a low‐grade intermittent fever. Worsening of these new symptoms over the following days led her to seek medical care.

On physical examination, the patient appeared acutely ill but was conscious and oriented. Her vital signs showed a blood pressure of 100/60 mmHg, tachycardia with an irregularly irregular pulse (radial pulse rate of 135 bpm, apical heart rate of 102 bpm), tachypnea (respiratory rate of 28 breaths per minute), a body temperature of 36.2°C, and an oxygen saturation of 93% on room air. Her weight was 42 kg and height was 1.55 m, resulting in a calculated body mass index of 17.5 kg/m^2^, indicating an underweight status.

She had pale conjunctiva and non‐icteric sclera but no angular cheilitis or glossitis. The anterior neck mass measured 2 × 2 cm. It was non‐tender, mobile, multinodular, firm, and not attached to the overlying skin, with no palpable lymphadenopathy. Chest examination revealed dullness on percussion and fine crepitations in the posterior bilateral lower one‐third. Additional findings included a raised jugular venous pressure, an active precordium, and a murmur consistent with mitral regurgitation. Abdominal examination was unremarkable, while grade one edema was present on the lower extremities. There were no deformities or limitations of movement. The patient's integumentary exam showed palmar and plantar pallor. She was alert and oriented to time, place, and person, with intact cranial nerves and normal motor and sensory examination. There were no signs of meningeal irritation.

## 3 Investigations, Diagnosis and Treatment

Laboratory results revealed severe hypochromic microcytic anemia with a hemoglobin level of 4.6 g/dL and a mean corpuscular volume of 64.1 fL. Other blood cell lines were within normal ranges, but her erythrocyte sedimentation rate was mildly elevated at 32 mm/h. Liver enzymes, coagulation profile, and bilirubin levels were normal, while serum albumin was low at 2.4 g/dL. Renal function tests were normal, but her serum electrolytes showed severe hypokalemia at 2.44 mmol/L. Cardiac enzymes were normal. Thyroid function tests revealed a very low TSH level of < 0.0001 mIU/L and an elevated free T4 level of 2.45 ng/dL. Urinalysis was unremarkable, and the stool occult blood test was positive (Table 1). Neck ultrasound confirmed a multinodular goiter, and fine‐needle aspiration cytology indicated a benign nodular colloid goiter. Chest x‐ray showed old fibrotic changes, and the cardiac silhouette appeared normal. The GeneXpert test for *Mycobacterium tuberculosis* in sputum was negative. Echocardiography revealed tachycardia with mild mitral regurgitation (MR) and mild aortic regurgitation (AR). There is biatrial enlargement with an absent A‐wave, consistent with chronic atrial fibrillation. Additionally, moderate tricuspid regurgitation (TR), a dilated inferior vena cava (IVC), and moderate pulmonary hypertension are noted. Taken together, these findings are consistent with thyro‐cardiac disease. Electrocardiography confirmed atrial fibrillation.

**TABLE 1:** Laboratory profile of the patient upon admission.

Variables	Lab. Values on Admission	References
*Complete blood count (CBC)*		
White blood cells (×10^3^/μL)	4.47	4.5–11.0
Hemoglobin (gm/dL)	4.6	12.0–15.0
Mean cell volume (fL)	64.1	80–100
Red blood cell width (%)	32.1	≤ 14%
Platelets (×10^3^/μL)	216	150–450
ESR (mm/h)	32	≤ 20
*Urinalysis*		
Protein	Negative	
Glucose	Negative	
Cast	Negative	
*Liver biochemical tests*		
ALT (IU/L)	10	≤ 40
AST (IU/L)	18	≤ 40
Bilirubin (total) (mg/dL)	0.8	0.3–1.0
Bilirubin (direct) (mg/dL)	0.2	0.03–0.18
Alkaline phosphatase (U/L)	68	34–104
Albumin (g/dL)	2.4	3.5–5.0
Total protein (g/dL)	5.5	6.4–8.9
PT (seconds)	13.5	12–14
APTT (seconds)	30.7	30–44
INR	1.3	0.830–1.290
*Renal function tests*		
Serum Cr (mg/dL)	0.4	0.6–1.3
Urea (mg/dL)	17	≤ 20
Serum Na (mmol/L)	133.5	135–145
Serum K+ (mmol/L)	2.44	3.5–5.5
*Thyroid function test*		
TSH (μU/mL)	< 0.0001	0.35–4.94
Free T3 (pg/dL)	2.45	1.58–3.91
Free T4 (ng/dL)	5.57	0.7–1.8
*Cardiac enzymes*		
Troponin (pg/L)	20.6	≤ 50

**FIGURE 1:**
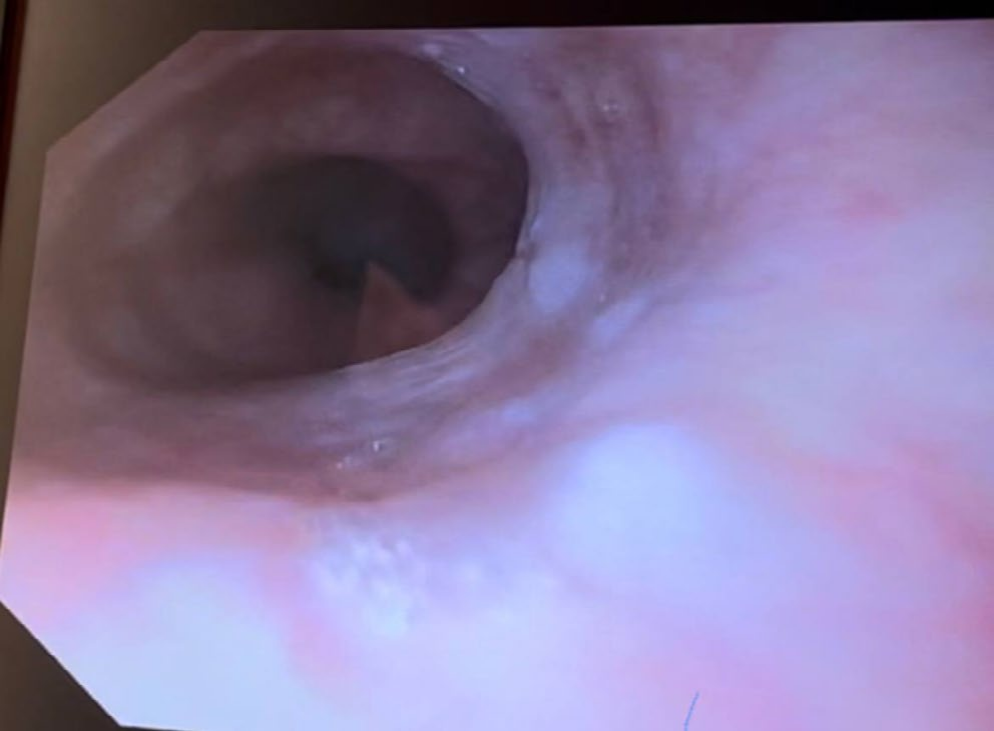
Upper GI endoscopy revealing esophageal webs.

**FIGURE 2:**
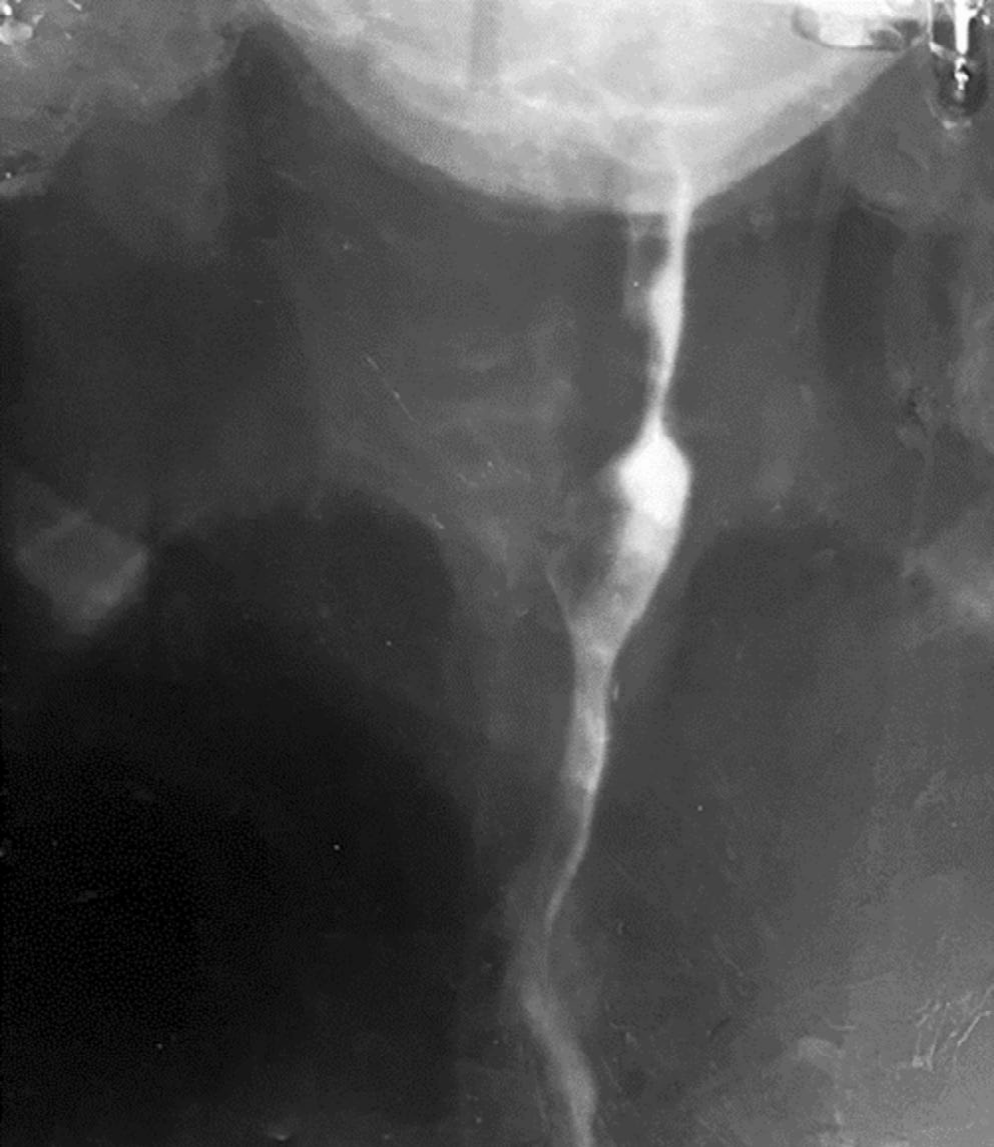
Barium swallow showing focal esophageal dilatation with an incomplete linear filling defect caused by the esophageal webs.

The patient was diagnosed with acute decompensated heart failure secondary to thyro‐cardiac disease precipitated by atrial fibrillation and anemia. Upper gastrointestinal endoscopy revealed esophageal webs and significantly reduced esophageal motility (Figure 1), raising suspicion for Plummer‐Vinson syndrome. A barium swallow showed focal esophageal dilatation with an incomplete linear filling defect caused by the esophageal webs (Figure 2).

The patient was initially treated with intravenous furosemide, potassium chloride, atenolol, propylthiouracil (PTU), and two units of whole blood. Following treatment, her symptoms of edema, orthopnea, and shortness of breath improved, and her potassium levels normalized. She was subsequently maintained on oral furosemide, PTU, and propranolol. For Plummer‐Vinson syndrome, the patient underwent pneumatic dilatation and was treated with PO iron sulfate. Deworming was also administered.

## 4 Outcome and Follow‐Up

The patient showed significant clinical improvement after treatment, with resolution of edema, orthopnea, and shortness of breath. Her dysphagia improved following pneumatic dilation and iron therapy. A follow‐up colonoscopy was unremarkable. Although iron studies could not be performed due to limited resources, the patient's overall condition stabilized. Regular follow‐up was recommended to monitor for potential complications, such as esophageal carcinoma, given the association with PVS.

## 5 Discussion

Patterson and Kelly first reported Plummer‐Vinson Syndrome (PVS) in 1919, consisting of a triad of dysphagia, iron deficiency anemia, and upper esophageal web(s) [1]. Most of the patients are middle‐aged women, in the fourth to seventh decade of life similar to our patient [3]. The pathogenesis of PVS is not fully understood but is believed to be related to iron deficiency, which leads to mucosal atrophy and the formation of esophageal webs [1, 2]. The diagnosis is typically made based on clinical presentation and confirmed by endoscopic findings of esophageal webs [1].

Iron deficiency anemia is a hallmark of PVS and is thought to contribute to the development of esophageal webs through mucosal atrophy and epithelial changes [4, 5]. The anemia in PVS is usually microcytic and hypochromic, as seen in this patient. The presence of esophageal webs can lead to dysphagia, which is often the presenting symptom [6]. In this case, the patient's dysphagia was progressive and involved both solid and liquid foods, consistent with the typical presentation of PVS. Enlargement of the spleen and thyroid is also observed [1].

The management of PVS involves addressing the underlying iron deficiency through iron supplementation and, in some cases, mechanical dilation of the esophagus to relieve dysphagia [7, 8]. Iron supplementation is crucial as it helps to correct the anemia and may lead to the resolution of esophageal webs [1]. In this case, the patient responded well to iron supplementation and pneumatic dilation, with significant improvement in swallowing.

PVS is associated with an increased risk of developing esophageal squamous cell carcinoma. Therefore, patients diagnosed with PVS should be monitored closely for any signs of malignancy. Regular follow‐up and endoscopic surveillance may be warranted to detect any early malignant changes [9].

The coexistence of thyro‐cardiac disease and PVS in this patient adds complexity to the clinical picture. Thyro‐cardiac disease, characterized by hyperthyroidism and cardiac manifestations, can lead to atrial fibrillation and heart failure, as seen in this patient [3]. The management of thyro‐cardiac disease involves controlling the hyperthyroidism and managing the cardiac complications. In this case, the patient was treated with PTU and beta‐blockers, which helped to stabilize her condition.

The presence of severe hypokalemia in this patient is also noteworthy. Hypokalemia can exacerbate cardiac arrhythmias and contribute to the development of heart failure [10]. The correction of hypokalemia was an important aspect of this patient's management and contributed to her clinical improvement.

In conclusion, this case highlights the importance of considering Plummer‐Vinson syndrome in patients presenting with dysphagia and iron‐deficiency anemia. Early diagnosis and appropriate management are crucial in preventing complications and improving patient outcomes. The coexistence of thyro‐cardiac disease and PVS in this patient emphasizes the need for a comprehensive and multidisciplinary approach to patient care.

## Author Contributions

**Suleiman Ayalew:** conceptualization, data curation, resources, writing – original draft. **Michael A. Negussie:** conceptualization, visualization, writing – review and editing. **Tinsae Anebo:** writing – review and editing. **Fitsum A. Gemechu:** writing – review and editing. **Yishak Abdulsemed:** writing – review and editing. **Segenet B. Mengistu:** resources, supervision.

## Consent

Written informed consent was obtained from the patient for the publication of this case report, including the presentation of clinical details, diagnostic images, and relevant medical information. The authors have ensured that all reasonable measures have been taken to protect the patient's privacy and confidentiality. No personally identifiable information has been included in the report. Consent to publish was acquired from all co‐authors.

## Conflicts of Interest

The authors declare no conflicts of interest.

## Data Availability

The data that support the findings of this study are available from the corresponding author upon reasonable request.
